# Evolution, Gene Duplication, and Expression Pattern Analysis of *CrRLK1L* Gene Family in *Zea mays* (L.)

**DOI:** 10.3390/ijms251910487

**Published:** 2024-09-29

**Authors:** Kai Wang, Baoping Xue, Yan He, Haibin Zhao, Bo Liu, Wenting Jiang, Pengfei Jin, Yanfeng Wang, Xiangqian Zhang, Xiaolong He

**Affiliations:** 1 Shaanxi Key Laboratory of Research and Utilization of Resource Plants on the Loess Plateau, College of Life Sciences, Yan’an University, Yan’an 716000, China; wangkai@yau.edu.cn (K.W.); xuebaoping@whu.edu.cn (B.X.); zhbyanandaxue@163.com (H.Z.); jiangwenting@yau.edu.cn (W.J.); wyf@yau.edu.cn (Y.W.); 2 Engineering Research Center of Microbial Resources Development and Green Recycling of Shaanxi Province, Yan’an University, Yan’an 716000, China; 13165776334@163.com (Y.H.); liubo4552@126.com (B.L.); jpf@yau.edu.cn (P.J.)

**Keywords:** *CrRLK1L* gene family, systematic evolution, duplication events, expression pattern

## Abstract

Catharanthus roseus receptor-like kinase 1-like (CrRLK1L) plays pivotal roles in regulating plant growth and development, mediating intercellular signal transduction, and modulating responses to environmental stresses. However, a comprehensive genome-wide identification and analysis of the *CrRLK1L* gene family in maize remains elusive. In this study, a total of 24 *CrRLK1L* genes were identified in the maize whole genome. A phylogenetic analysis further revealed that CrRLK1L proteins from *Arabidopsis*, rice, and maize were grouped into nine distinct subgroups, with subgroup IV being unique to maize. Gene structure analysis demonstrated that the number of introns varied greatly among *ZmCrRLK1L* genes. Notably, the genome-wide duplication (WGD) events promoted the expansion of the *ZmCrRLK1L* gene family. Compared with *Arabidopsis*, there were more collinear gene pairs between maize and rice. Tissue expression patterns indicated that *ZmCrRLK1L* genes are widely expressed in various tissues, with *ZmCrRLK1L5/9* specifically highly expressed in roots, and *ZmCrRLK1L8/14/16/21/22* expressed in anthers. Additionally, RNA-seq and RT-qPCR analyses revealed that the expression of *ZmCrRLK1L1/2/20/22* genes exhibited different expression patterns under drought and salt stresses. In summary, our study lays a foundation for elucidating the biological roles of *ZmCrRLK1L* genes in maize growth and development, reproductive development, and stress responses.

## 1. Introduction

Receptor-like kinases (RLKs), which constitute the largest receptor family in plants, play a pivotal role in perceiving and transducing diverse environmental stimuli [1]. Among them, the CrRLK1L (Catharanthus roseus RLK1-like kinase) receptor kinase family, a unique subfamily exclusive to plants, has garnered considerable attention in recent research [2,3,4]. *CrRLK1L* family members are typically located on the cytoplasmic membrane, possess kinase activity, and participate in intracellular and extracellular signal communication by recognizing various ligands (such as RALF peptides, etc.) [5,6]. Furthermore, CrRLK1Ls also have transmembrane domains and intracellular kinase domains, with the latter being responsible for translating extracellular signals into intracellular signals, thereby regulating the growth and development of plants [7].

CrRLK1L receptors play crucial roles in plant growth, development, immunity, and fertilization. Recently, multiple *CrRLK1L* family members have been identified in *Arabidopsis* and rice, exhibiting diverse expression patterns and functions across tissues and developmental stages [8,9]. These receptors recognize specific ligands, including RALF peptides, to activate downstream signaling, thereby modulating plant stress responses, such as drought and salt tolerance. For instance, OsRALF45/46 regulates drought tolerance and induces ROS production in rice mediated by OsMRLK63 [10], while LRX proteins sense cell wall changes under salt stress to release RALF proteins, enhancing salt tolerance [11]. After RALF1 binds to FER, it activates downstream MAPK cascades, enhancing plant salt tolerance [12].

In recent years, CrRLK1L-RALF interaction has emerged as one of the central focuses in plant biology research. For instance, the FERONIA receptor kinase, through recognizing RALF, is involved in regulating root development and growth, impacting root cell elongation and division by recognizing RALF1 [13,14,15,16,17]. The ANX/BUPS receptor kinase complex modulates pollen tube integrity and sperm release by recognizing the RALF4/19 peptides secreted by the pollen tube itself and the RALF34 peptide secreted by female tissues [18]. Moreover, in *Arabidopsis*, the binding of RALF23 to FER initiates the MAPK cascade, fostering ROS production and cell wall reinforcement, thereby enhancing plant disease resistance [19,20,21]. Moreover, LLG2/3, as a co-receptor of the BUPS-ANX receptor complex, is involved in the recognition of RALF4/19 and RALF34 peptides and regulates the cell integrity of *Arabidopsis* pollen tubes [22].

Maize is a globally critical crop and staple food source. Moreover, extreme temperatures, drought, and high salt stresses affect approximately 90% of cultivable areas, resulting in 70% yield reductions of significant food crop of maize, sorghum, rice, and wheat [23]. Given *CrRLK1L*’s pivotal role in plant growth, development, and stress responses, elucidating its functions in food crop is crucial for enhancing environmental stress resistance [10,11,12]. Additionally, *CrRLK1L* gene families have been identified in various plants [8,9]. However, the functions of CrRLK1L proteins are currently poorly understood in maize, especially in response to abiotic stresses. Therefore, this study aims to comprehensively analyze the *ZmCrRLK1L* gene family in maize. In this study, we identified 24 *ZmCrRLK1L* in the maize genome and conducted a systematic analysis of their phylogenetic relationships, protein motifs, gene structures, duplication events, cis-acting elements, and expression patterns across tissues and abiotic stress conditions. These results facilitate further exploration of the regulatory mechanisms of *CrRLK1L* family members in maize under abiotic stresses.

## 2. Results

### 2.1. Identification of ZmCrRLK1L Family Genes

**Figure 1:**
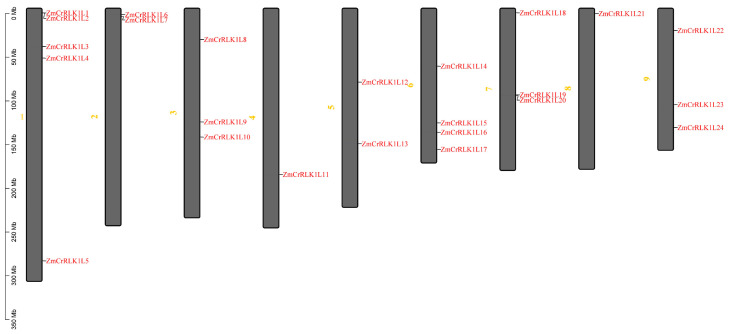
Locations of the *ZmCrRLK1L* genes on the chromosome of maize. The yellow numbers represent different chromosomes numbers.

In this study, we identified 24 *CrRLK1L* members in the maize genome (Supplementary Table S1). Subsequently, these *ZmCrRLK1L* genes were named *ZmCrRLK1L1*–*ZmCrRLK1L24* based on their chromosomal locations (Figure 1). Furthermore, we analyzed the physicochemical properties of the ZmCrRLK1L family proteins. As shown in Supplementary Table S1, the amino acid sequence lengths of ZmCrRLK1L proteins vary from 474 aa (ZmCrRLK1L2) to 1225 aa (ZmCrRLK1L17), and their molecular weights range from 51.46 to 133.28 kD. The isoelectric point (pI) of the ZmRALF family proteins is from 5 (ZmCrRLK1L14) to 8.41 (ZmCrRLK1L3). Specifically, ZmCrRLK1L2, 3, 13, and 23 are acidic proteins, while the remainder are basic proteins. Based on the instability index analysis of the ZmRALF family proteins, we found that ZmCrRLK1L1/2/6/9/10/12/17 are stable proteins, whereas the other members belong to unstable proteins with an instability index greater than 40. Moreover, we found that the ZmCrRLK1L2 protein is hydrophobic, whereas the other proteins are hydrophilic, with a grand average of hydropathicity less than zero.

### 2.2. Phylogenetic Analysis of ZmCrRLK1L, OsCrRLK1L, and AtCrRLK1L Proteins

**Figure 2:**
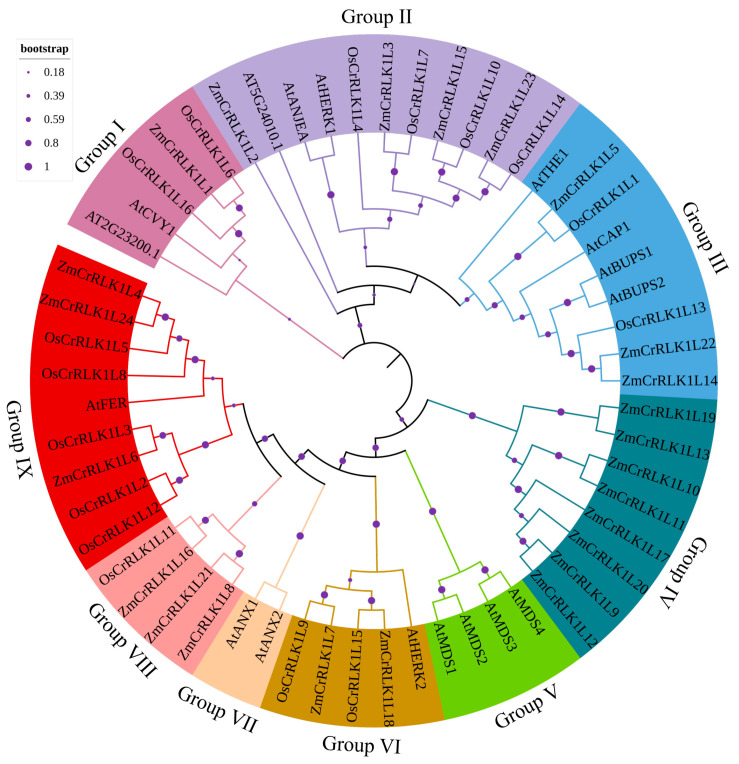
An evolutionary tree was constructed based on the ZmCrRLK1L, OsCrRLK1L, and AtCrRLK1L protein sequences. This evolutionary tree was generated through MEGA 7.0 software (method: neighbor-joining; bootstrap: 1000). Different colors represent different subgroups.

To understand the evolutionary relationships among ZmCrRLK1L, OsCrRLK1L, and AtCrRLK1L proteins, we constructed a phylogenetic tree based on 24 ZmCrRLK1L, 16 OsCrRLK1L, and 17 AtCrRLK1L proteins. As shown in Figure 2, these CrRLK1L proteins can be divided into nine subfamilies: Group I, Group II, Group III, Group IV, Group V, Group VI, Group VII, Group VIII, and Group IX. We found that AtMADS1-AtMADS1 and AtANX1-AtANX1 were specific to Group V and Group VII, respectively, while Group IV is specifically present in maize. *Arabidopsis* receptor kinase Feronia (FER) is a star member of the CrRLK1L subfamily of plant receptor kinases that plays an important role in the three physiological processes of reproduction, growth, and immunity. Interestingly, we found that ZmCrRLK1L4 and ZmCrRLK1L14 are closely related to the evolution of AtFER, suggesting that ZmCrRLK1L4 and ZmCrRLK1L14 may also play an important role in these biological processes and deserve further investigation in the future. A recent study showed that BUPS1 and BUPS2 are necessary for normal growth of pollen tubes in the pistil [21]. Notably, *OsCrRLK1L13*, *ZmCrRLK1L14*, *ZmCrRLK1L22*, *BUPS1*, and *BUPS2* are grouped into the same subgroup, suggesting that *ZmCrRLK1L14*, *ZmCrRLK1L22*, and *OsCrRLK1L13* may also have similar functions.

### 2.3. Analysis of Gene Structure and Conserved Protein Motifs

**Figure 3:**
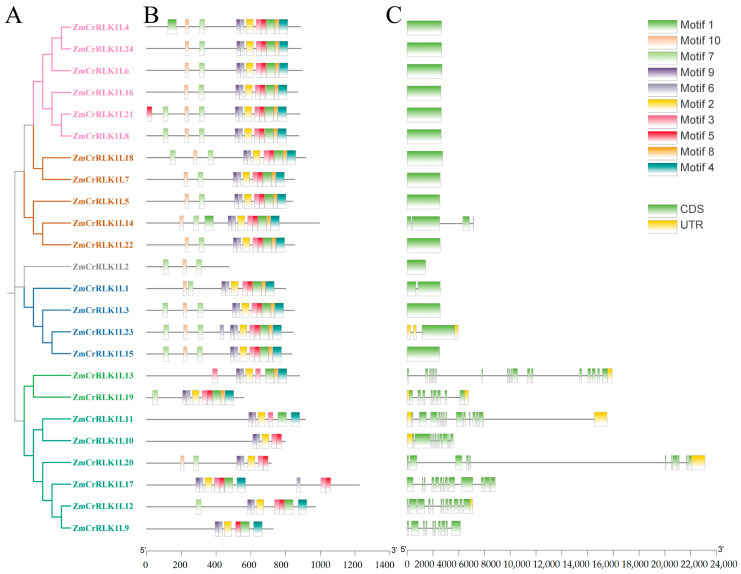
The gene structure and conserved protein motifs of ZmCrRLK1L. (**A**) An evolutionary tree was constructed based on the ZmCrRLK1L protein sequences. This evolutionary tree was generated through MEGA 7 software (method: neighbor-joining; bootstrap: 1000). (**B**) Conserved protein motifs were predicted using the online website MEME. (**C**) The gene structure was analyzed based on the GFF file. This image was visualized using Tbtools v2.121 (Beijing, China) [25].

To further elucidate the evolutionary relationships of *ZmCrRLK1L* family members, we conducted a comprehensive analysis of gene structures and conserved protein motifs. As shown in Figure 3, our findings reveal a notable phenomenon: approximately half of the *ZmCrRLK1L* family members lack introns, whereas the remaining half harbor multiple introns, with their numbers varying significantly from 1 to 17. This observation is consistent with previous studies on eggplant [24]. These results suggest that *ZmCrRLK1L* has undergone functional differentiation during evolution.

Furthermore, we predicted the conserved motif distribution of ZmCrRLK1L proteins and identified ten conserved motifs (Figure 3). Notably, motifs 2 through 10 are ubiquitously present in the majority of *ZmCrRLK1L* family members, suggesting their fundamental roles in maintaining structural or functional integrity. However, an intriguing exception is observed in ZmRALF2/3/4/5/10/13, which notably lacks motif 2, while motif 1 is exclusively present in ZmCrRLK1L4 (Figure 3), pointing to specific evolutionary trajectories and potential functional diversification. Intriguingly, our investigation also uncovered a correlation between intron content and motif composition (Figure 3). Specifically, ZmCrRLK1L members with a higher number of introns were found to be devoid of motifs 5, 7, 8, and 10. This finding implies that the presence or absence of these motifs, in combination with intron content, may be indicative of distinct functional roles or evolutionary pressures shaping the ZmCrRLK1L family.

### 2.4. Replication Event Analysis of the ZmCrRLK1L Gene Family

**Figure 4:**
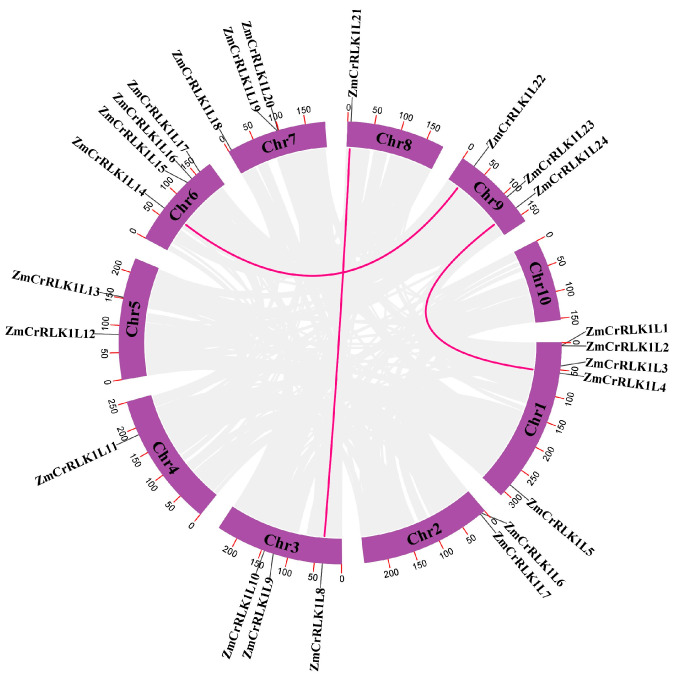
Duplication event analysis of *ZmCrRLK1L* gene. The pink line represents tandem replication gene pairs.

To gain insight into the expansion pattern of the *ZmCrRLK1L* gene family, we analyzed the duplication events of the *ZmCrRLK1L* gene family using MCScan X (Beijing, China). Our investigation revealed the presence of three distinct whole-genome duplication (WGD) gene pairs of *ZmCrRLK1L* with the maize genome, while no tandem duplication (TD) events were observed (Figure 4). These findings underscore the primary role of WGD in driving the expansion of the *ZmCrRLK1L* gene family in maize, aligning well with previous studies conducted on *T. aestivum* [26], tomato [27], and eggplant [24]. Furthermore, by analyzing the ka/ks ratios, we discovered that all duplication gene pairs exhibited values less than 1 (Supplementary Table S2). This observation signifies that the *CrRLK1L* gene family in maize has undergone purifying selection during its expansion process.

### 2.5. Collinearity Relationship Analysis of ZmCrRLK1L Genes

**Figure 5:**
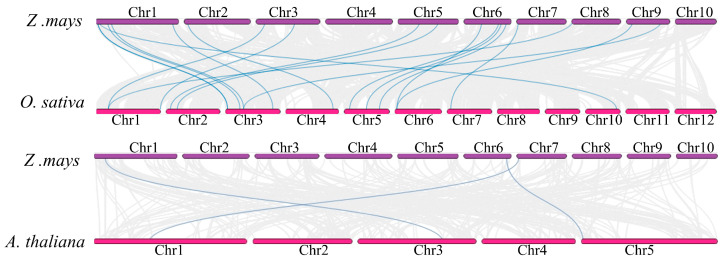
Collinear gene pair analysis of *ZmCrRLK1L* between maize and rice and *Arabidopsis*. The blue line represents collinear gene pairs.

To gain deeper insight into the evolutionary mechanism of the *ZmCrRLK1L* gene family, we analyzed the collinear gene pairs between maize and rice and *Arabidopsis* based on McScan. Our analysis revealed the existence of 19 collinear gene pairs between maize and rice and 3 collinear gene pairs between maize and *Arabidopsis* (Figure 5). This observation underscores the potential for greater functional conservation among *ZmCrRLK1L* genes within maize and rice, in contrast to the more pronounced functional divergence evident between *ZmCrRLK1L* and *AtCrRLK1L* genes. Notably, maize exhibited the highest number of collinear *ZmCrRLK1L* gene pairs with rice, emphasizing the evolutionary proximity of these two species. It is particularly noteworthy that the *ZmRALF5/10/14* genes possess syntenic counterparts in both rice and *Arabidopsis*, suggesting a shared ancestral origin for these genes. This finding highlights the significance of these *CrRLK1L* family members in the evolutionary history of these plant species. Intriguingly, *ZmCrRLK1L8/15* formed two distinct syntenic gene pairs with *ZmCrRLK1L13*, indicating a complex evolutionary interplay between specific *CrRLK1L* family members in maize.

### 2.6. Analysis of Cis-Acting Elements of ZmCrRLK1L Gene

**Figure 6:**
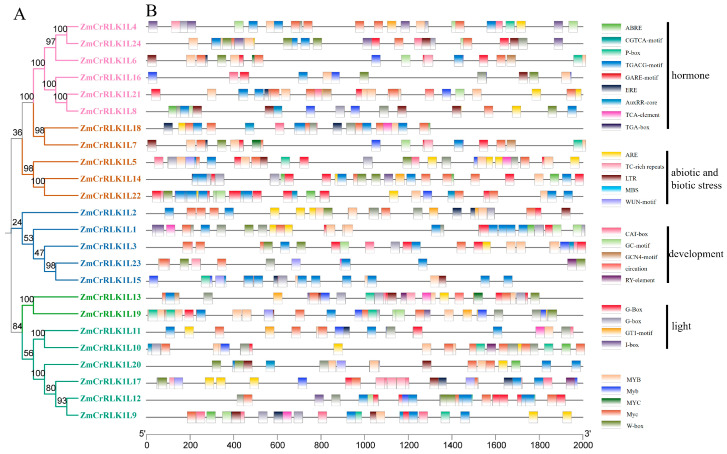
Cis-acting elements were predicted for *ZmCrRLK1L* gene promoter sequences. (**A**) An evolutionary tree was constructed based on the ZmCrRLK1L protein sequences. This evolutionary tree was generated through MEGA 7 software (method: neighbor-joining; bootstrap: 1000). (**B**) The cis-acting elements were predicted for *ZmCrRLK1L* gene 2 kb promoter sequences. These cis-acting elements include hormone, abiotic and biotic, growth and development, and light and transcription factor binding sites.

To explore the potential biological functions of the *ZmCrRLK1L* family genes, we used Plant-CARE to predict cis-acting elements in the promoters of *ZmCrRLK1L* genes. We identified 28 major types of cis-regulatory elements, including abscisic acid (ABA), methyl jasmonate (MeJA), auxin (IAA), gibberellin (GA), salicylic acid (SA), drought (MBS), low temperature (LTR), circadian, meristem, endosperm, defense, and stress responsiveness (Figure 6 and (Supplementary Table S3). Furthermore, we observed that most of the promoter regions of the *ZmCrRLK1L* family genes include W-box, MYB, and MYC cis-elements, which are typically associated with stress, suggesting that the *ZmCrRLK1L* gene may play an important role in coping with environmental stresses. Notably, the *ZmCrRLK1L1/5/9/11/13/18* genes were found to contain JA and SA cis-acting elements, suggesting a potential antagonistic role in regulating the JA and SA signaling pathways (Figure 6). Additionally, the *ZmCrRLK1L2/6/7/9/11/15/17/18/20/21* genes exhibited the presence of JA and ET-related cis-elements, implying a potential synergistic regulation of the JA and ET signaling cascades by these genes (Figure 6). In summary, our findings highlight *ZmCrRLK1L* potential involvement in modulating plant responses to diverse environmental stimuli, particularly through intricate crosstalk among hormone signaling pathways.

### 2.7. Expression Pattern Analysis of ZmCrRLK1L Genes in Different Tissues

**Figure 7:**
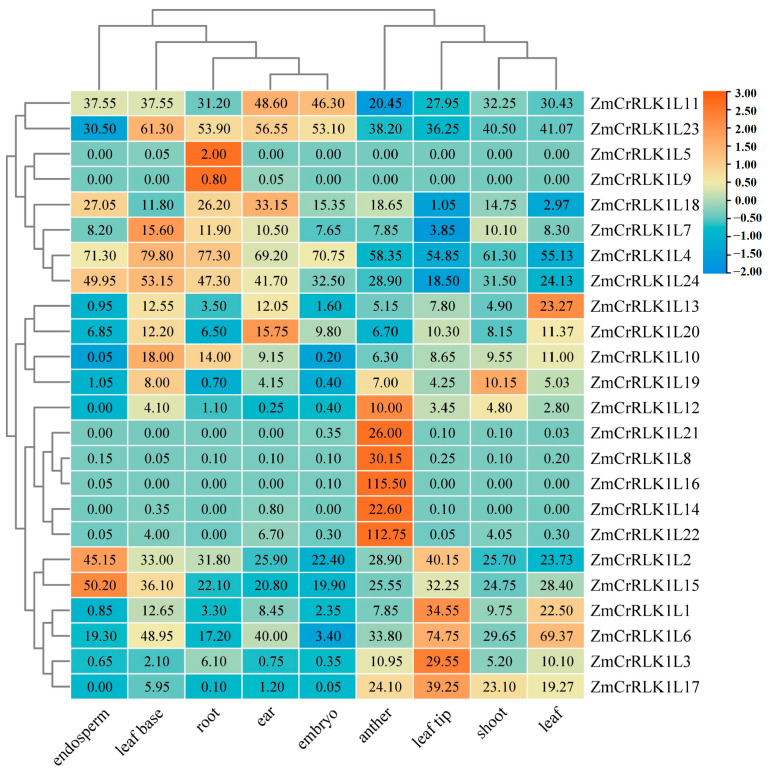
Expression pattern analysis of ZmCrRLK1L in different tissues. Expression pattern of ZmCrRLK1L in different tissues including root, endosperm, leaf base, ear, embryo, anther, leaf tip, shoot, and leaf. Red and blue boxes indicate high and low expression levels of ZmCrRLK1L genes.

To further reveal the potential function of *ZmCrRLK1L* genes, we analyzed their expression patterns across eight tissues, including root, leaf base, leaf tip, shoot, anther, leaf, ear, endosperm, and embryo (Supplementary Table S4). As shown in Figure 7, most of the *ZmCrRLK1L* genes are widely expressed in various tissues, while some are highly expressed in specific tissues. For example, *ZmCrRLK1L8*, *ZmCrRLK1L14*, *ZmCrRLK1L16*, *ZmCrRLK1L21*, and *ZmCrRLK1L22* in anther; *ZmCrRLK1L2* and *ZmCrRLK1L15* in endosperm; *ZmCrRLK1L5* and *ZmCrRLK1L19* in root; and *ZmCrRLK1L1*, *ZmCrRLK1L3*, *ZmCrRLK1L6*, and *ZmCrRLK1L17* in leaf tip. Notably, the presence of a cis-element related to endosperm expression in the promoter region of the *ZmCrRLK1L2* gene, suggests that *ZmCrRLK1L2* may be involved in maize endosperm development. Previous studies have shown that ANX1/2 and BUPS1/2 are both members of the CrRLK1L receptor family and are mainly expressed in pollen tubes, forming receptor complexes that regulate the cell integrity and spermatocytes release of pollen tubes by recognizing RALF4/19 and RALF34 [28]. Given the specific high expression of *ZmCrRLK1L8/14/16/21* in anther, we hypothesize that these genes may play a crucial role in pollen tube development.

### 2.8. Expression Patterns Analysis of ZmCrRLK1L Genes under Different Abiotic Stresses

**Figure 8:**
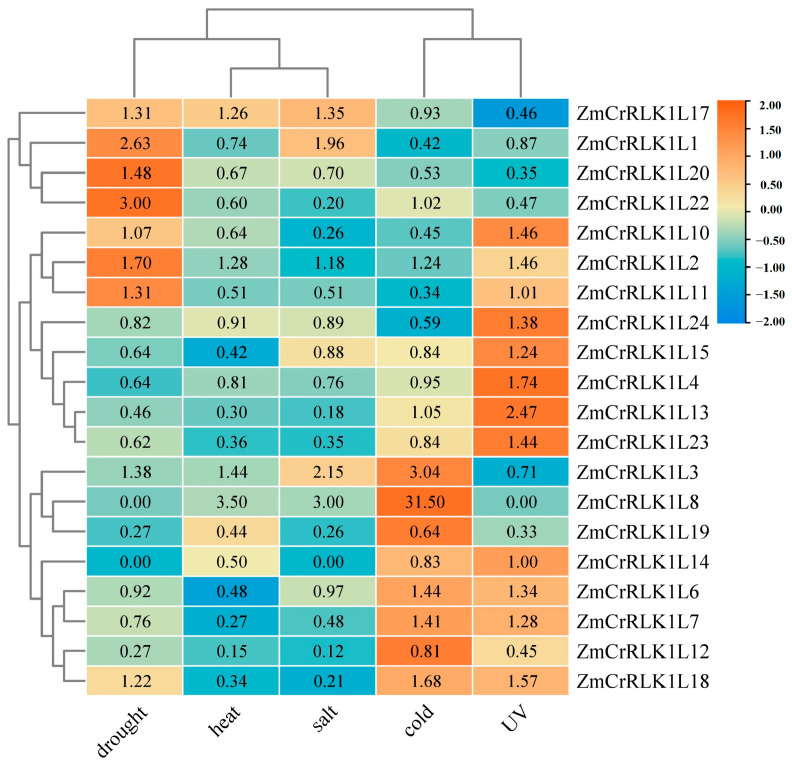
The expression pattern analysis of ZmCrRLK1L in different abiotic stresses. The expression pattern of ZmCrRLK1L in different abiotic stresses including drought, heat, salt, and cold. Red and blue boxes indicate high and low expression levels of ZmCrRLK1L genes.

Environmental stresses adversely impact plant growth and development, prompting plants to mount diverse physiological, molecular, biochemical, and genetic responses. Among these, *CrRLK1L* genes have been shown to play a pivotal role in plant abiotic stress responses, as supported by previous studies [10]. In this study, we performed a comprehensive analysis of *ZmCrRLK1L* gene expression profiles under various stress conditions, utilizing RNA-seq data. Our results revealed that *ZmCrRLK1L* genes exhibited differential expression patterns under drought, heat, salt, and cold stresses (Supplementary Table S5). Specifically, we observed significant upregulation of 8, 4, 3, and 6 *ZmCrRLK1L* genes, respectively, and downregulation of 6, 11, 9, and 5 genes, correspondingly, under drought, heat, salt, and cold stresses. (Figure 8). Notably, *ZmCrRLK1L8* is a highly responsive gene, exhibiting marked upregulation by 31.5-fold, 3.5-fold, and 3-fold under cold, heat, and salt stresses, respectively. Conversely, *ZmCrRLK1L7*, *ZmCrRLK1L12*, *ZmCrRLK1L13*, *ZmCrRLK1L19*, and *ZmCrRLK1L23* exhibited varying degrees of downregulation under drought, heat, and salt stresses. In contrast, *ZmCrRLK1L1*, *ZmCrRLK1L2*, *ZmCrRLK1L20*, and *ZmCrRLK1L22* were upregulated under both heat and salt stresses, hinting their potential involvement in specific stress response pathways (Figure 8). Interestingly, *ZmCrRLK1L8* and *ZmCrRLK1L14* were not induced under drought stress, but upregulated and downregulated, respectively, under heat stress.

### 2.9. Expression Pattern Analysis of ZmCrRLK1L1/2/20/22 under PEG and Salt Treatments by RT-qPCR

**Figure 9:**
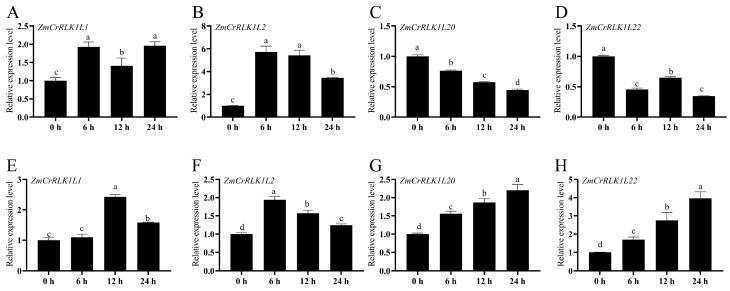
Expression patterns of *ZmCrRLK1L* genes under abiotic stress by RT-qPCR. (**A**–**D**) Expression levels of *ZmCrRLK1L1*, *ZmCrRLK1L2*, *ZmCrRLK1L20*, *and ZmCrRLK1L22* genes under 200 mM NaCl. (**E**–**H**) Expression levels of *ZmCrRLK1L1*, *ZmCrRLK1L2*, *ZmCrRLK1L20*, *and ZmCrRLK1L22* genes under 20% PEG6000 treatment. For the PEG and NaCl treatments, the maize seedlings were cultured with Hoagland nutrient solution for the three-leaf stage, then transferred into normal solution, 20% PEG6000, and 200 mM NaCl, respectively. Samples of roots were obtained and collected at 0 h, 6 h, 12 h, and 24 h after initiation of the stress treatment. X-axis represents stress treatment time points, and Y-axis represents the relative expression level. The expression levels were calculated using 2^−△△Ct^ methods. The gene expression at 0 h was set to 1, and relative expression level in the other time points was relative to 0 h. The data are expressed the standard error (SE) based on three replicates. Different letters indicate significant differences by one-way analysis of variance (ANOVA). RT-qPCR analysis was performed using the *Zm00001d013367* gene as an internal control.

To further explore the potential function of *ZmCrRLK1L* genes, we selected four *ZmCrRLK1L* genes that exhibited responsiveness to both drought and salt stress conditions. We quantitatively analyzed their differential expression patterns using RT-qPCR after treatment with 20% PEG6000 (mimicking drought) and 200 mM NaCl (simulating salt stress), respectively (Figure 9). Notably, *ZmCrRLK1L1*, *ZmCrRLK1L2*, *ZmCrRLK1L20*, and *ZmCrRLK1L22* exhibited significant upregulation in 20% PEG6000 treatment, peaking at 12 h, 6 h, 24 h, and 24 h, respectively (Figure 9). In contrast, under 200 mM NaCl treatment, the expression of *ZmCrRLK1L1* and *ZmCrRLK1L2* increased, whereas *ZmCrRLK1L20* and *ZmCrRLK1L2*2 displayed a downregulation. Intriguingly, *ZmCrRLK1L20* and *ZmCrRLK1L22* showed opposing expression trends between the two stress treatments (Figure 9). Specifically, their expression levels were upregulated following 20% PEG6000 treatment but downregulated upon salt stress treatment, highlighting the distinct regulatory mechanisms underlying their responses to different environmental cues. This finding underscores the complexity and specificity of the stress-responsive pathways involving *ZmCrRLK1L* genes, suggesting that they may play crucial, yet divergent, roles in mediating plant adaptation to drought and salt stress.

## 3. Discussion

Plant receptor kinase CrRLK1L plays a pivotal role in reproduction, growth, and immunity [2,3,4,10,13,14,15,16]. However, understanding of the function of *CrRLK1L* family genes in maize is still lacking. Therefore, we comprehensively used bioinformatics to analyze the physicochemical properties, conserved motifs, duplication events, and expression patterns of *ZmCrRLK1L* genes. In this study, 24 *ZmCrRLK1L* members were found in the maize genome (Figure 1). *CrRLK1L* is widely found in plants, of which 17, 16, 24, 38, 26, and 43 are identified in *Arabidopsis*, rice, tomato, soybean, *C. quinoa*, and *T*. *aestivum*, respectively [26,27,29,30,31,32]. Compared to other species, the number of *ZmCrRLK1L* members is lower than in *T*. *aestivum* and soybean but higher than in *Arabidopsis* and rice. The genome of *T*. *aestivum* consists of three subgenomes (A, B, and D), the formation of which involves two distant hybridization and allopolyploidy processes of the three ancestral species [33]. Soybean, a typical paleopolyploid, underwent two rounds of genome-wide replication events, resulting in nearly 75% of genes being in multiple copy form [34]. We speculate that the reason may be that wheat is a typical allohexaploid crop, soybean is a typical paleopolyploid plant with a complex and large genome, while *Arabidopsis* has only five chromosomes and a small and simple genome.

Gene duplication is one of the most important driving forces of genome evolution, and it is also one of the primary reasons for the emergence of genes with novel functions and the evolution of new species [35]. In the process of species evolution, the original gene of one copy in the ancestral species evolves into a gene with multiple copies following WGD [36]. Tandem duplication is closely associated with the amplification of genes related to biotic and abiotic stresses [37]. Since plants differ from animals, when they encounter environmental changes or attacks by external organisms, plants cannot escape like animals. Therefore, a stress response has evolved in plant life activities to counteract the damage caused by the environment and external organisms [38]. In our study, three WGD gene pairs were found in *ZmCrRLK1L*, suggesting that the *ZmCrRLK1L* gene family is primarily replicated by WGD during its expansion process. Collinearity analysis revealed intriguing insights into the evolutionary relationships among species. Specifically, we observed 18 homologous gene pairs shared between maize and rice, indicating a high degree of conservation in their *CrRLK1L* gene functions. In contrast, when compared to *Arabidopsis*, only three homologous gene pairs were observed, suggesting a potentially more pronounced functional divergence in the *CrRLK1L* gene family between maize and *Arabidopsis*.

Previous studies have found that CrRLK1L not only regulates plant root development, reproduction, and hypocotyl elongation, but also plays an important role in plant immune response and plant response to abiotic stress [10,13,14,15,16]. On the one hand, FER is closely related to *ZmCrRLK1L14*; on the other hand, *ZmCrRLK1L1*4 is specifically highly expressed in anthers, suggesting that *ZmCrRLK1L14* plays an important role in the reproductive development of maize. HERK1 and ANJ are localized in unfertilized ovules; most of them remain unfertilized due to pollen tube overgrowth, suggesting that HERK1 and ANJ mediate the interaction between male and female gametophytes during plant fertilization [19]. Given that ZmCrRLK1L3, ZmCrRLK1L15, and ZmCrRLK1L23 cluster in the same subgroup as ANJ, we speculate that *ZmCrRLK1L3*, *ZmCrRLK1L15*, and *ZmCrRLK1L23* may play a role in the fertilization process of maize. However, maize is a cross-pollinated plant, and *Arabidopsis* is a self-pollinated plant; thus, there may be functional differentiation between the *CrRLK1L* genes in maize and *Arabidopsis*.

BUPS1/BUPS2 proteins, in concert with ANX1 and ANX2 complexes, maintain the integrity of the pollen tube and prevent it from breaking before reaching the synergid cells [28]. Notably, ANX1/2 is closely related to ZmCrRLK1L16/18/21, and BUPS1/2 is in the same subgroup as *ZmCrRLK1L14/22*, suggesting that these genes are homologs genes of maize *ANX* and *BUPS* in *Arabidopsis* (Figure 2). This result indicates that *ZmCrRLK1L16/18/21* and *ZmCrRLK1L14/22* may play an important role in pollen fertilization. This conclusion was supported by tissue-specific gene expression patterns, where *ZmCrRLK1L16/18/21* and *ZmCrRLK1L14/22* display heightened expression levels in the anther (Figure 7). However, it is imperative to acknowledge that this conclusion is limited. Future studies should encompass phenotypic assessments to fortify our understanding of the precise mechanisms by which *ZmCrRLK1L* family members contribute to the intricate process of pollen tube guidance and fertilization in maize.

Furthermore, our findings revealed that the promoter sequences of the *ZmCrRLK1L3* and *ZmCrRLK1L8* genes harbor low-temperature-responsive elements, whereas the promoter regions of the *ZmCrRLK1L20* and *ZmCrRLK1L22* genes contain drought-responsive elements. Additionally, we observed that the expression levels of *ZmCrRLK1L3* and *ZmCrRLK1L8* genes were significantly upregulated under cold stress, while *ZmCrRLK1L20* and *ZmCrRLK1L22* were enhanced under drought stress conditions. These observations suggest that these genes may play pivotal roles in mediating plant adaptation to drought and cold stress, respectively. Our hypotheses are corroborated by previous research conducted in rice [10] and *Arabidopsis* [12], lending credence to the potential functional significance of these genes in stress tolerance mechanisms.

## 4. Materials and Methods

### 4.1. Identification and Phylogenetic Analysis of ZmCrRLK1L Family Genes in Maize

The maize whole-genome sequence and genome annotation files were downloaded from phytozome 13 [39]. The HMM (hidden Markov model) of Malectin_like (PF12819) and Pkinase_Tyr (PF07714) were obtained from the Pfam database [40]. Seventeen AtCrRLK1L and sixteen OsCrRLK1L protein sequences were downloaded from TAIR (https://www.arabidopsis.org/, accessed on 18 June 2024) [41] and the Rice Genome Annotation Project (http://rice.uga.edu/, accessed on 18 June 2024), respectively. Firstly, using AtCrRLK1L and OsCrRLK1L protein sequences as a reference, the BLASTP method was used to alignment the sequences with maize protein sequences (E-value < 1 × 10^−5^), respectively. Secondly, we constructed a hidden Markov model to search for potential ZmCrRLK1L proteins by HMM 3.0 software (E-value < 1 × 10^−5^). Then, after the removal of duplicates and integration of the maize, CrRLK1L was obtained via the above two procedures. Finally, candidate ZmCrRLK1L protein sequences were submitted to NCBI-CDD (https://www.ncbi.nlm.nih.gov/Structure/bwrpsb/bwrpsb.cgi, accessed on 20 June 2024) to confirm the conserved RALF domain.

MEGA 7 (method: neighbor-joining; bootstrap number: 1000) software was used to construct a phylogenetic tree of ZmCrRLK1L, AtCrRLK1L, and OsCrRLK1L protein sequences [42]. Lastly, the picture of phylogenetic tree was embellished through the online website iTOL (https://itol.embl.de/, accessed on 20 June 2024).

### 4.2. Gene Structure, Protein Motif, and Cis-Acting Element Analysis of ZmCrRLK1L

The gene structures and protein conserved motifs of ZmCrRLK1L were analyzed by genome annotation file and MEME [43], respectively. The 2 kb promoter sequence of *ZmCrRLK1L* gene was submitted to PlantCARE (http://bioinformatics.psb.ugent.be/webtools/plantcare/html/, accessed on 21 June 2024) to predict potential cis-regulatory elements [44]; then, the picture was visualized based on the Tbtools v2.121 (Beijing, China) software.

### 4.3. Orthologous Gene, Paralogous Gene, and Ka/KS Ratio Analysis of ZmCrRLK1L

The tandem duplication and whole-genome duplication gene pairs were analyzed by MCscan X (Beijing, China) software [45]. The ka, ks, and ka/ks analyses were performed by Tbtools v2.121 (Beijing, China) software. The orthologous gene (between maize and rice and *Arabidopsis*) was analyzed by McScan software [45].

### 4.4. The ZmCrRLK1L Gene Expression Patterns Analysis

The tissue expression profiles of the *ZmRALF* gene were analyzed from various tissues downloaded from PPRD (http://ipf.sustech.edu.cn/pub/plantrna/, accessed on 29 June 2024) (PRJEB35943) [46]. The maize breed was a B73 inbred maize line; these tissues were collected from various tissues and at different development stages, including embryo, endosperm, anther, ear, leaf tip, leaf, leaf base, shoot, and root. The transcriptome data of *ZmCrRLK1L* gene in various abiotic stresses, such as salt (PRJNA244661), cold (PRJNA244661), heat (PRJNA244661), and drought (PRJNA378714) were downloaded from PPRD.

### 4.5. Plant Materials, Growth Conditions, and PEG and NaCl Treatments

Maize cultivar B73 inbred maize line was used in this study. The maize seeds were disinfected with 75% ethanol for 1 min, and then washed with distilled water 5 times to remove the ethanol. The sterilized seeds are evenly spread in the seedling pots, covered with vermiculite, and irrigated with distilled water to make vermiculite absorb enough water [47]. After three days of germination, the seeds were transferred to black boxes containing Hoagland nutrient solution [47], and the Hoagland nutrient solution was replaced every three days. Seedlings of the maize were cultivated in a greenhouse under conditions of 14/10 h light/dark (200 µmol m^−2^ s^−1^) and 27 °C.

For the PEG and NaCl treatments, the maize seedlings were cultured with Hoagland nutrient solution for the three-leaf stage, then transferred into normal solution, 20% PEG6000, and 200 mM NaCl, respectively [47]. Samples of roots were obtained and collected at 0 h, 6 h, 12 h, and 24 h after initiation of the stress treatment [47]. This treatment concentration and sampling time point refers to the method of Zhu et al [47].

### 4.6. Total RNA Extraction and RT-qPCR Analysis

In our study, we used primer 5.0 (Canada) software to design *ZmCrRLK1L* gene-specific RT-qPCR primer (Supplementary Table S6). Total RNA from maize roots was extracted by E.Z.N.A.^®^ Plant RNA Kit (Omega, Norcross, GA, USA). The RNA concentration was examined by means of a BioDrop Cambridge, UK ultraviolet nucleic acid assay. The cDNA was a reverse transcription synthesis by PrimeScript™ RT reagent Kit (Perfect Real Time) (TaKaRa, Nojihigashi, Japan). The first-strand cDNA was reverse-transcribed from 1000 ng total RNA, with a volume of 20 µL and then diluted four times to be used for RT-qPCR. The RT-qPCR assays were performed using a real-time PCR analyzer (CFX384 Touch Real-Time PCR Detection System (Bio-Rad, Hercules, CA, USA). Each RT-qPCR reaction mixture contained  5 µL SYBR, 3 µL No RNA enzyme water, 1 µL cDNA sample, 0.5 µL forward primer (final concentration 10 μM), and 0.5 µL reverse primer (final concentration 10 μM) in a final volume of 10 µL. RT-qPCR amplification program: 95 °C (5 min), then 45 cycles of 95 °C (15 s) and 60 °C (1 min). Melting curve analysis was used to verify the specificity of the reaction, and the melting curve analysis results are shown in Supplementary Figure S1.

### 4.7. Statistical Analysis

The expression levels were calculated using 2-^△△Ct^ methods. The data are expressed the standard error (SE) based on three replicates. Different letters indicate significant differences by one-way analysis of variance (ANOVA). The gene expression at 0 h was set to 1, and relative expression level in the other time points was relative to 0 h. RT-qPCR analysis was performed using the *Zm00001d013367* gene as an internal control. The X-axis represents stress treatment time points, and the Y-axis represents the relative expression level.

## 5. Conclusions

In summary, 24 *CrRLK1L* genes were identified in the maize whole-genome and divided into nine subgroups. WGD was the main driving force of *CrRLK1L* gene family expansion in maize and might have undergone intense purifying selection during the evolutionary process. Moreover, the *CrRLK1L* gene has a large variation in intron number between different subgroups. Furthermore, RT-qPCR and RNA-seq results indicated that *ZmCrRLK1L1/2/20/22* may have an important role in drought and salt stresses. Our research data provide valuable resources for future functional characterization and verification of the *CrRLK1L* maize gene.

## Data Availability

Data are contained within the article or Supplementary Materials.

## Supplementary Materials

The following supporting information can be downloaded at: https://www.mdpi.com/article/10.3390/ijms251910487/s1.
