# Supplementary figures and images for: Evolution, Gene Duplication, and Expression Pattern Analysis of CrRLK1L Gene Family in Zea mays (L.)
Source: Int J Mol Sci. 2024 Sep 29;25(19):10487. doi: 10.3390/ijms251910487 (PMC11477507; doi:10.3390/ijms251910487)

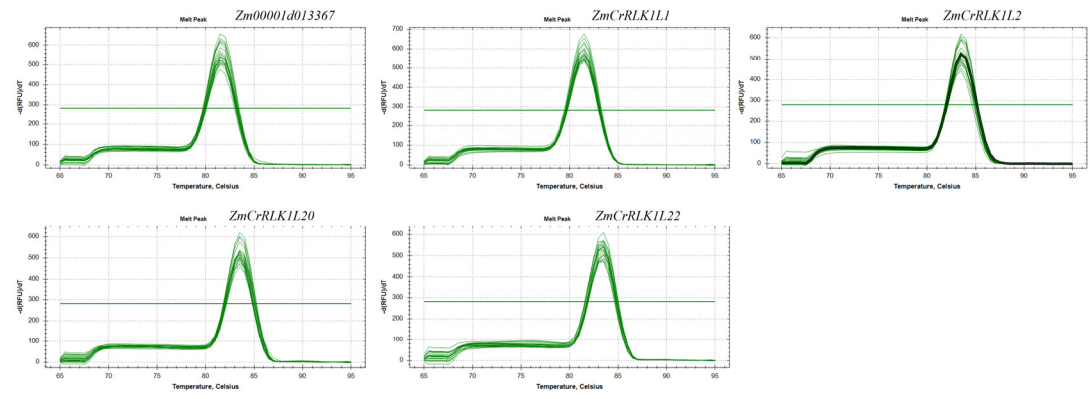

**Figure S1.** The melting curve analysis of *Zm00001d013367*, *ZmCrRLK1L1*, *ZmCrRLK1L2*, *ZmCrRLK1L20* and *ZmCrRLK1L22*.

Supplement: Supplementary file 1 [file ijms-25-10487-s001.zip › Supplemental Figure S1.pdf]
